# Prebiotic Supplementation has Only Minimal Effects on Growth Efficiency, Intestinal Health and Disease Resistance of Westslope Cutthroat Trout *Oncorhynchus clarkii lewisi* Fed 30% Soybean Meal

**DOI:** 10.3389/fimmu.2015.00396

**Published:** 2015-08-27

**Authors:** Wendy M. Sealey, Zachariah B. Conley, Molly Bensley

**Affiliations:** ^1^Bozeman Fish Technology Center, U.S. Fish and Wildlife Service, Bozeman, MT, USA; ^2^Department of Animal and Range Sciences, Montana State University, Bozeman, MT, USA; ^3^Bozeman Fish Health Center, U.S. Fish and Wildlife Service, Bozeman, MT, USA

**Keywords:** cutthroat trout, soybean meal enteritis, *Flavobacterium psychrophilum*, prebiotic, disease resistance

## Abstract

Prebiotics have successfully been used to prevent infectious diseases in aquaculture and there is an increasing amount of literature that suggests that these products can also improve alternative protein utilization and digestion. Therefore, the objective of this study was to examine whether prebiotic supplementation increased the growth efficiency, intestinal health, and disease resistance of cutthroat trout fed a high level of dietary soybean meal. To achieve this objective, juvenile Westslope cutthroat trout (*Oncorhynchus clarkii lewisi*) were fed a practical type formulation with 0 or 30% dietary soybean meal with or without the commercial prebiotic (Grobiotic-A) prior to experimental exposure to *Flavobacterium psychrophilum*. Juvenile Westslope cutthroat trout (initial weight 7.8 g/fish ±SD of 0.5 g) were stocked at 30 fish/tank in 75 L tanks with six replicate tanks per diet and fed their respective diets for 20 weeks. Final weights of Westslope cutthroat trout were affected by neither dietary soybean meal inclusion level (*P* = 0.9582) nor prebiotic inclusion (*P* = 0.9348) and no interaction was observed (*P* = 0.1242). Feed conversion ratios were similarly not affected by soybean meal level (*P* = 0.4895), prebiotic inclusion (*P* = 0.3258) or their interaction (*P* = 0.1478). Histological examination of the distal intestine of Westslope cutthroat trout demonstrated increases in inflammation due to both increased soybean meal inclusion level (*P* = 0.0038) and prebiotic inclusion (*P* = 0.0327) without significant interaction (*P* = 0.3370). Feeding dietary soybean meal level at 30% increased mortality of *F. psychrophilum* cohabitation challenged Westslope cutthroat trout (*P* = 0.0345) while prebiotic inclusion tended to decrease mortality (*P* = 0.0671). These results indicate that subclinical alterations in intestinal inflammation levels due to high dietary inclusion levels of soybean meal could predispose Westslope cutthroat trout to *F. psychrophilum* infection.

## Introduction

Prebiotics are defined as non-digestible food ingredients that beneficially affect the host by stimulating growth and/or activity of a limited number of health-promoting bacteria in the intestine while limiting potentially pathogenic bacteria (1). Prebiotics have successfully been used to treat infectious diseases in aquaculture [(2, 3); for reviews see Ref. (4, 5)]. More recently, there is an increasing amount of literature that suggests that these products can also improve digestion of dietary nutrients and energy in some fish species, increasing feed efficiency (6, 7) increasing growth (8, 9), and tempering stress responses (10, 11) as reviewed by Ringo et al. (12). Importantly, beneficial effects often have been observed when fish were cultured under less than optimum environmental conditions (13), when fish are exposed to pathogenic organisms (14) or when salmonids are fed alternative proteins containing anti-nutrients (15, 16).

Confounding additive effects of anti-nutrients in alternative proteins on gastrointestinal health have been associated with observations of reduced growth and feed efficiency in salmonids. Specifically, a wealth of information exists on detrimental effects of soybean meal on salmonid intestinal health and microbiota structure (17–24). In soybean meal-induced enteritis, the intestinal tract becomes inflamed as characterized by increased mucosal leukocyte accumulations, epithelial cell proliferation and finally enteric diarrhea when the intestine becomes inflamed (25). Decreases in immunocompetence have also been observed and are believed to be linked to the crucial immunogenic role of the hindgut of teleost fish in helping to keep the organism healthy (26, 27). However, a growing body of evidence suggests that host-microbial interactions also may result in dys-regulated mucosal immune responses (28). Even when gamete quality, genetic selection, physiochemical conditions, and nutritional requirements are well controlled or kept constant under hatchery conditions, variability among replicates has been hypothesized to be the result of detrimental fish–microbe interactions in the gut (29). The beneficial effect(s) of prebiotics in animals fed alternative protein sources also have been attributed to their ability to bind and inactivate plant antigens of glycoprotein nature and/or promoting growth of beneficial bacteria in the gastrointestinal tract of terrestrial animals (30, 31). Thus, microbial manipulations to improve fish health and alternative protein utilization using prebiotics have great potential even though how they mediate host benefits at the mucosal level is still poorly understood (32).

*Flavobacterium psychrophilum*, a gram-negative, filamentous, psychrotrophic bacterium belonging to the phylum Bacteroidetes, is the causative agent of bacterial cold-water disease (BCWD) and rainbow trout fry syndrome. *F. psychrophilum* is considered one of the most important salmonid pathogens worldwide (33) because of the severe mortalities caused by infection with this pathogen and the resulting economic impact among commercial aquaculture producers and conservation hatcheries (34). Laboratory studies have shown that BCWD can be spread horizontally from fish to fish (35, 36). However, it is believed that horizontal transmission is much greater when there is a predisposing condition that damages the mucosal barriers of the skin (36). More recent research by Evenhuis and Cleveland (37) has demonstrated that other mucosal barriers also display upregulated immune responses during *F. psychrophilum* infection. In that study, measurable changes in gene expression for a number of innate immune molecules were up-regulated in intestinal tissue of rainbow trout following intramuscular injection challenge with *F. psychrophilum* (37).

Previous research in our laboratory demonstrated that normal growth rates in Snake River cutthroat trout (*Oncorhynchus clarkia behnkei*) fed 30% soybean meal in a 10-week feeding trial (38). However, the histopathology data in that study demonstrated soy-level-dependent negative effects on gut health similar to that previously observed for juvenile trout (39, 40). Because the gastrointestinal damage of cutthroat trout previously observed in our laboratory could alter the crucial role of physical and immunological barrier that trout gastrointestinal mucosal surfaces could play in horizontal transmission of BCWD, the purpose of the current study was to examine whether prebiotic supplementation increased the growth efficiency, intestinal health and thereby disease resistance of cutthroat trout fed 30% dietary soybean meal.

## Materials and Methods

### Experimental animals

Juvenile Westslope cutthroat trout (approximately 4 g initial weight) were obtained from Montana Department of Fish, Wildlife and Parks’ Washoe Park Trout Hatchery, Anaconda, Montana. Fish were stocked into 200-L tanks, supplied with 14°C flow-through spring water and fed a commercially produced 2-mm sinking feed (Classic Fry; Skretting North America, Tooele, UT, USA) until feeding trial initiation. All fish were handled and treated in accordance with the USFWS procedures according to the Guidelines for Use of Fishes in Research (41).

### Diet formulation and production

A practical-type diet was formulated to meet or exceed the nutrient requirement of juvenile rainbow trout (42, 43). The diet was formulated to contain approximately 41% digestible protein and 15% crude lipid with fish meal, and chicken concentrate meal as the primary protein sources (Table 1). Test diets were formulated to include 0 or 30% soybean meal (SBM) with reciprocal reductions in fish meal and chicken concentrate on an equivalent digestible protein basis using rainbow trout digestible protein values (44) and 0 or 2% functional yeast product (Grobiotic-A, International Ingredient Company). All diets were supplemented with crystalline methionine, lysine, and threonine to the ideal amino acid targets of 3.8, 1.3, and 2.1%, respectively; total phosphorus was brought to 1.2% through the addition of monocalcium phosphate. Prior to mixing, all ingredients were ground using an air-swept pulverizer (Jacobsen 18H, Minneapolis, MN, USA). Dry ingredients were mixed in a horizontal paddle mixer (Marion Mixers, Marion, Iowa) and a portion (~1/3) of the added oil was mixed into the dry ingredients along with the lecithin. The mash was then extruded through a 3.0-mm die of a Buhler twin-screw cooking extruder (DNDL-44, Buhler AG, Uzwil, Switzerland). Barrel temperature averaged 124°C in Sections 2–6. Die pressure varied from 34 to 47 bar (3,400–4,700 kPa), depending on diet. Feed had a barrel residence time of approximately 13 s. Diets were dried in a pulse bed drier extruder (Buhler AG, Uzwil, Switzerland) with air discharge temperature remaining below 104°C, and final moisture content <8%. After diets were dried, they were top-coated with the remaining oil at ambient pressure, and stored at room temperature (~18–23° C).

**Table 1:** **Composition (% dry matter) of the experimental formulation fed to juvenile Westslope cutthroat trout**.

Ingredient	Fish meal (0% SBM)		Soybean meal (30% SBM)	
Soybean meal^a^	0.0	0.0	30.0	30.0
Grobiotic-A	0.0	2	0.0	2
Wheat flour^d^	31.35	29.35	13.95	11.95
Sardine meal^c^	25.0	25.0	12.0	12.0
Chicken concentrate^b^	17.0	17.0	8.0	8.0
Menhaden fish oil^e^	8.2	8.2	9.3	9.3
Poultry fat	0.0	0.0	1.6	1.6
Corn protein concentrate^f^	4.0	4.0	4.0	4.0
Spirulina^g^	3.0	3.0	3.0	3.0
Squid meal^h^	3.0	3.0	3.0	3.0
Lecithin	2.0	2.0	2.0	2.0
Stay-C 35^i^	0.2	0.2	0.2	0.2
Vitamin premix ARS^j^	1.0	1.0	1.0	1.0
TM ARS 640^k^	0.1	0.1	0.1	0.1
Astaxanthin^l^	0.05	0.05	0.05	0.05
Taurine^m^	0.5	0.5	0.5	0.5
Choline Cl 50%	1.0	1.0	1.0	1.0
Monocalcium phosphate	1.0	1.0	3.0	3.0
DL-Methionine	0.5	0.5	0.85	0.85
Lysine HCl	1.6	1.6	3.2	3.2
Threonine	0.5	0.5	1.25	1.25

*^a^Archer Daniels Midland Company, 472 g/kg crude protein*.

*^b^IDF Inc., 832 g/kg protein*.

*^c^Peruvian prime sardine, Skretting, 649 g/kg crude protein*.

*^d^Manildra Milling, 120 g/kg protein*.

*^e^Omega Proteins Inc., Virginia Prime menhaden oil*.

*^f^Cargill Inc., Empyreal 75, 756 g/kg crude protein*.

*^g^Earthrise, 620 g/kg crude protein*.

*^h^Antarctic Sea Fisheries, F/V Betanzos, 638 g/kg crude protein*.

*^i^Stay-C, 35%, DSM Nutritional Products*.

*^j^Contributed per kg of diet: vitamin A (as retinol palmitate), 30,000 IU; vitamin D_3_, 2160 IU; vitamin E (as dl-%-tocopheryl-acetate), 1590 IU; niacin, 990 mg; calcium pantothenate, 480 mg; riboflavin, 240 mg; thiamin mononitrate, 150 mg; pyridoxine hydrochloride, 135 mg; menadione sodium bisulfate, 75 mg; folacin, 39 mg; biotin, 3 mg; vitamin B_12_, 90 μg*.

*^k^Contributed in mg/kg of diet: zinc, 37; manganese, 10; iodine, 5; copper, 3; selenium, 0.4*.

*^l^DSM Nutritional Products, Carophyl Pink 10 (10% active astaxanthin)*.

*^m^NB Group Co. LTD*.

### Growth trial

Westslope cutthroat trout had an initial weight of 7.8 g/fish (±0.5 g) and were counted into groups (30 fish) and placed into 75-L tanks. The four treatments were randomly allocated, with six replicate tanks per treatment, for a total of 24 experimental tanks. Each tank was supplied with constant temperature water (14°C ± 2) at a flow rate of 8 L/min. Photoperiod was maintained at a constant 13 h light and 11 h dark with fluorescent lighting. Fish in the trial were bulk-weighed and counted every 4 weeks, and growth rates and feed conversion ratios were calculated according to the following formulae:

$$\begin{array}{l} \text{Weight gain (}\%\text{ increase)}=\frac{\text{Final fish weight (g)}-\text{initial fish weight (g)}}{\text{Initial fish weight (g)}}\times 100\\ \text{Feed conversion ration (FCR)}=\frac{\text{Feed intake [dry weight (g)]}}{\text{Weight gain [wet weight (g)]}} \end{array}$$

At the conclusion of the feeding trial, fish from each tank were randomly selected and the gastrointestinal tract was examined histologically for cellular changes related to dietary modifications. Two fish from each of the six replicate tanks were euthanized and a section measuring approximately 2 cm was dissected from the anterior portion of the distal intestine of each fish. Samples were preserved in Dietrich’s solution until processed by standard histological procedures (45).

Since feeding of high levels of soybean meal is known to cause severe lesions in the distal intestine of Atlantic salmon, and to a lesser degree rainbow trout, histological evaluation focused on the anterior portion of the distal intestine. Two sections, taken at different depths, of intestine were examined for each fish. Based on results of previous diet studies, sections were evaluated for general cellular changes and the presence of the following alterations: inflammation and increased thickness of the stratum granulosum layer, lamina propria, or connective tissue. Observed cellular changes recorded for each fish were scored on a scale of 0–4: 0 = no change; 1 = minimal; 2 = mild; 3 = moderate; and 4 = marked. In all specimens the presence of fecal material during preservation and processing resulted in tissue artifact that prevented an adequate evaluation of mucosal fold height or supranuclear vacuolization in mucosal epithelial cells.

### Disease trial

After 20 weeks of feeding of respective diets, a subsample of remaining fish from each tank were experimentally exposed to live *F. pyschrophilum*; Twenty fish were randomly selected and 10 fish in each tank had their dorsal fins clipped and were subcutaneously injected with 10 μL of *F. psychrophilum* suspended in 0.85% saline for a dose of 6.25 × 10^7^ cfu/fish (46). This challenge protocol produces a clinical disease similar in presentation to a natural infection and has been reported as an appropriate model infection (47). The remaining 10 fish in each tank were sham injected with 10 μL of 0.85% saline to assess effects of infection due to cohabitation. During the challenge trial, Westslope cutthroat trout were cultured as previously described and mortalities and/or moribund fish were recorded daily for 42 days post challenge (14 days post-cessation of mortality). A minimum of 20% of the daily mortalities were re-examined and confirmed positive by detection of *F. psychrophilum* in kidney imprints using the FITC-labeled MAb FL43.

### Statistical analyses

The PROC MIXED procedure, SAS Software Version 7.00 (SAS institute, Inc., Cary, NC, USA) was used to conduct a factorial analysis of variance for a mixed effects model (48) in which soy level and prebiotic inclusion were defined as fixed effects and tanks within treatments were defined as a random effect. For all analyses, binomial data were transformed using arcsine transformation prior to analysis. Differences within main effects were determined using the Tukey procedure for pair-wise comparisons (49).

## Results

### Growth

Final weights of Westslope cutthroat trout were affected by neither dietary soybean meal inclusion level (*P* = 0.9582) nor prebiotic inclusion (*P* = 0.9348) and no interaction was observed (*P* = 0.1242; Table 2). Westslope cutthroat trout growth increase ranged from 380 to 400% with no significant effects of soybean meal level (*P* = 0.4526), prebiotic inclusion (*P* = 0.8480), or their interaction (*P* = 0.5295). Feed conversion ratios were similarly not affected by soybean meal level (*P* = 0.4895), prebiotic inclusion (*P* = 0.3258) or their interaction (*P* = 0.1478). Survival of Westslope cutthroat trout fed all diets was acceptable, ranging from 73 to 86%, and was not altered by soybean meal level (*P* = 0.5451), prebiotic inclusion (*P* = 0.2022), or their interaction (*P* = 0.4384).

**Table 2:** **Growth performance and survival of Westslope cutthroat trout fed diets containing 0 or 2% Grobiotic-A with 0 or 30% soybean meal (SBM) for 20 weeks**.

Diets	Weight gain [final average fish wt (g)]	Weight gain (% increase)	Feed conversion ratio	Survival (%)
0% SBM
0% Grobiotic-A	39.6	387	1.0	86
2% Grobiotic-A	37.1	380	1.1	80
30% SBM
0% Grobiotic-A	37.1	388	1.2	73
2% Grobiotic-A	39.4	400	1.0	79
Pooled SE	1.5	14	0.08	3.2
*P*-values
SBM	0.9582	0.4526	0.4895	0.5451
Grobiotic-A	0.9348	0.8480	0.3258	0.2022
Grobiotic-A X SBM	0.1242	0.5295	0.1478	0.4384

### Intestinal health

Histological examination of the distal intestine of Westslope cutthroat trout fed 30% soybean meal for 20 weeks demonstrated statistically significant increases in distal intestine inflammation due to both increased soybean meal inclusion level as denoted by uppercase letters in Table 3 (*P* = 0.0038) and due to prebiotic inclusion as denoted by lower case letters in Table 3 (*P* = 0.0327) without significant interaction between the factors (*P* = 0.3370). However, no pathological lesions were observed in any fish examined. Generally histological scores of 1 and 2 are considered normal or cellular changes of no significance; 3 is transitional or intermediary, moderate cellular changes that may or may not be within the normal range depending on species, age, and sex of the fish; and 4 is indicative of pathological lesions.

**Table 3:** **Distal intestine inflammation of Westslope cutthroat trout fed diets containing 0 or 2% Grobiotic-A with 0 or 30% soybean meal (SBM) for 20 weeks**.

Diets	Inflammation score
0% SBM
0% Grobiotic-A	0.33Bb
2% Grobiotic-A	0.67Ba
30% SBM
0% Grobiotic-A	0.92Ab
2% Grobiotic-A	1.75Aa
Pooled SE	0.25
*P*-values
SBM	0.0038
Grobiotic-A	0.0327
Grobiotic-A X SBM	0.3370

*Values within columns with a common letter do not differ significantly at P < 0.05; upper case letters refer to a significant soybean meal effect while lower case letters refer to significant prebiotic effect; no significant interactions were observed*.

### Disease trial

A dietary soybean meal level of 30% resulted in a significant increase in the mortality of cohabitated Westslope cutthroat trout (*P* = 0.0345) while prebiotic inclusion only tended (*P* = 0.0671) to decrease mortality of cohabitated fish following disease challenge (Table 4). However, no statistically significant effect of dietary soybean meal or prebiotic inclusion on Westslope cutthroat mortality following *F. psychrophilum* injection challenge was detected. Additionally, no significant interactive effects between soybean meal level and prebiotic supplementation on survival following experimental exposure to *F. psychrophilum* were observed.

**Table 4:** **Mortality (%) of Westslope cutthroat trout fed diets containing 0 or 2% Grobiotic-A with 0 or 30% soybean meal (SBM) for 20 weeks and then exposed to *Flavobacterium psychrophilum***.

Diets	Injected fish	Cohabitated fish
	Mortality	Mortality
0% SBM
0% Grobiotic-A	25.0	1.7B
2% Grobiotic-A	41.7	0.0B
30% SBM
0% Grobiotic-A	48.9	6.9A
2% Grobiotic-A	40.0	2.0A
Pooled SE	9.4	1.6
*P*-values
SBM	0.2477	0.0345
Grobiotic-A	0.6442	0.0671
Grobiotic-A X SBM	0.2022	0.3560

*Values within columns with a common letter do not differ significantly at P < 0.05; upper case letters refer to a significant soybean meal effect; no significant prebiotic effects or interactions were observed*.

## Discussion

The lack of growth enhancement in Westslope cutthroat trout with prebiotic supplementation agrees with previous research by Sealey et al. (50) who demonstrated no beneficial effects of Grobiotic-A supplementation on growth and feed efficiency of rainbow trout. However, these results contrast with more recent studies that investigated the effects of inclusion of Grobiotic-A in rainbow trout by Azari et al. (51, 52) who reported significantly improved specific growth rate, condition factor, and protein efficiency ratio with prebiotic supplementation. Although reasons for these differences are likely multifactorial, the substantial differences in the control diets dietary protein composition and notably, the high level of fish meal used by Sealey et al. (50) may be one explanation. Plant protein diets have been reported to alter the gastrointestinal microbiota of fish and alteration in their composition has been suggested as possible cause of reduced growth in these aquatic animals (53–55). Thus, an increased level of plant-based protein sources in the studies by Azari et al. (51, 52) likely increased the ability of the prebiotic supplement to improve growth relative to the control diet in that study. Lending support for this theory is the significant increase in the anaerobic and lactic acid bacteria observed in the gastrointestinal microbial community that was detected by Azari et al. (51, 52).

In the current study, even when a more conservative fish meal inclusion level and increased plant protein inclusion (commodity grade soybean meal containing anti-nutrients at the NRC reported levels) were applied, no benefit of prebiotic supplementation on growth efficiency of cutthroat trout was detected. It is possible that the additive effects of the formulation strategy of the present study may have minimized the beneficial effects of prebiotic supplementation by minimizing shifts in gastrointestinal microbial communities previously observed when rainbow trout were fed high levels of soybean meals (39). Of note, the formulation strategy of the current study, which included the use of highly palatable ingredients provided on equivalent digestible protein levels, increased supplementation of methionine, lysine, and threonine (56), fortification of vitamins at higher than NRC (57) requirements (58), macro-mineral and inositol supplementation (59), and taurine supplementation (56) has been shown to improve rainbow trout growth performance when fed plant-based diets. Because gastrointestinal microbial community was not investigated in the current study confirmation of this theory is impossible; however, previous work in our laboratory examining the effects of dietary protein blends on gastrointestinal microbial communities supports this hypothesis (23).

The lack of effect of soybean meal on growth of cutthroat trout recapitulates the results of our previous study in cutthroat trout (38). It is generally accepted that low to moderate levels of soybean meal may be included in salmonid diets without serious negative effects on growth or feed utilization (60, 61) as long as diets are formulated to meet the fish’s nutritional needs, but inflammatory responses of the gut still will be present (62). The soybean levels investigated in the current study demonstrated soybean meal-level-dependent negative effects on gut health similar to that previously observed for juvenile rainbow trout (39, 40) and more recently in cutthroat trout in our laboratory (38). Iwashita et al. (63) attributed similar effects on gut histology to soybean lectins and saponins when these anti-nutrients were included at a level equivalent to 50% SBM inclusion even though growth rate was not impaired. Thus, the inability of prebiotic supplementation to reduce the soybean meal-induced intestinal histological effects may indicate an inability of the tested prebiotic (Grobiotic-A), which is described as a mixture of partially autolyzed brewer’s yeast, dairy components, and dried fermentation products, to bind and inactivate soybean meal antigens in contrast to the mechanism described for the more purified mannan oligosaccharides-based prebiotic examined by Newman (30).

Feeding Westslope cutthroat trout dietary soybean meal at 30% also significantly increased mortality due to *F. psychrophilum* following cohabitation challenge. Previously in salmonids, increased susceptibility to furunculosis (60) in soybean meal-fed fish has been linked to suppressed immune capacity (17, 25, 64). Makesh et al. (65) recently proposed that mucosal association of IgT and IgD contribute to immediate protection of rainbow trout following anal intubation exposure to an attenuated strain of *F. psychrophilum*. Thus, it is possible that soybean meal-induced immunosuppression in the current study may have altered expression of IgT and IgD in the mucosa of the intestine and reduced survival.

The non-significant tendency (*P* = 0.0671) toward a beneficial effects of prebiotic supplementation on cutthroat trout survival following *F. psychrophilum* cohabitation challenge in the present study is in agreement with Sealey et al. (50) who reported that dietary Grobiotic-A improved survival of rainbow trout after experimental challenge with infectious hematopoietic necrosis virus. However, the effects of Grobiotic-A following pathogen exposure also have previously been investigated in a variety of fish species with various pathogens often with conflicting results (3, 13, 66–70). It is thus unclear whether the lack of significant effects following injection challenge in the present study alludes to the inability of the injection challenge models to account for the importance of mucosal barrier integrity in BCWD transmission as has previously been proposed (35, 36) and/or bypass the prebiotic benefits of gut microbial shifts and inhibition of pathogens (71). The latter mode of prebiotic action has been described previously in rainbow trout fed Grobiotic-A (51, 52).

Taken together these preliminary data seem to suggest that subclinical alterations in intestinal inflammation levels due to high dietary inclusion levels of soybean meal can predispose Westslope cutthroat trout to *F. psychrophilum* infection. Additional corroborating research utilizing additional response variables is necessary to identify the putative mechanism(s) and methods to mitigate these effects.

## Conflict of Interest Statement

The authors declare that the research was conducted in the absence of any commercial or financial relationships that could be construed as a potential conflict of interest.
